# China’s Successful Recruitment of Healthcare Professionals to the Worst-Hit City: A Lesson Learned

**DOI:** 10.3390/ijerph18168737

**Published:** 2021-08-19

**Authors:** Pingting Zhu, Xinyi Liu, Qiwei Wu, Jennifer Loke, Deborah Lim, Huiwen Xu

**Affiliations:** 1 Nursing School, Yangzhou University, 136 Jiangyang Middle Road, Yangzhou 225009, China; MX120190879@yzu.edu.cn (X.L.); MZ120201760@yzu.edu.cn (Q.W.); 006721@yzu.edu.cn (H.X.); 2 Jiangsu Key Laboratory of Zoonosis, 136 Jiangyang Middle Road, Yangzhou 225009, China; 3 Faculty of Health Sciences, University of Hull, Cottingham Road, Hull HU67RX, UK; 4 Park View Surgery, 87 Beverley Road, Hessle HU13 9AJ, UK; deborah.lim@nhs.net

**Keywords:** COVID-19, medical doctors, nurses, professional values, qualitative

## Abstract

The outbreak of coronavirus disease in 2019 (COVID-19) in Wuhan has led Chinese health authorities to recruit healthcare providers from the least-affected areas to provide care to the infected patients in Wuhan. We took further steps to explain some plausible reasons for their experiences. We used interpretative phenomenological analysis (IPA) to understand the subjective experiences, as well as the reasons for these experiences among the healthcare providers who had traveled from the least-affected parts of China to render aid during Wuhan’s COVID-19 outbreak. Using purposive and snowball sampling, healthcare professionals were recruited from three major hospitals in Jiangsu province. Semi-structured interviews were conducted from 1 September to 14 November 2020 in face-to-face contexts. Ten nurses and four doctors provided their informed consent for the study. The primary superordinate theme from the responses highlighted how social identity and individual needs were challenged by each individual’s professional ethics. COVID-19 not only presents significant risks to the health of nurses and medical doctors; it further challenges their emotional and psychosocial wellbeing. Care should be taken in allocating support and help, with the careful deployment of professional values and beliefs, so that any human resource as precious as medical doctors and nurses can be protected.

## 1. Introduction

The 2019 novel coronavirus, abbreviated as 2019-nCoV or COVID-19, was first identified in China in the year 2019. COVID-19 has sent a shockwave across the entire globe, except in Antarctica; by early 2020, many parts of the world had begun to experience major political, scientific and public health challenges. Lockdowns and borders closing to both international and national travel, despite a fast-approaching financial recession, were unavoidable strategies for many countries. Still, the responses from different governments varied. Between January 2020 and March 2020, when the world death toll for COVID-19 was rising sharply, discussions by the British Medical Association focused on the balance between personal protective equipment supply and medical services availability [1]. At the same time, concerted efforts by the Royal College of Nursing in the United Kingdom were centered around building resources for nurses providing patient care [2]. The ongoing discussions regarding nurses’ remuneration were intensified during the pandemic period [3]. At that point in time, the health authorities in China were occupied with a very different agenda; they were busy recruiting their very first batch of medical doctors and nurses from the less infectious regions to combat the virus in the worst-affected city of Wuhan, Hubei [4]. During the recruitment, China had already reported an estimate of 3000 health care workers infected by the disease [5]. By February 2020, the confirmed cases of infection among healthcare professionals had risen to 1716 with 6 deaths [6]. In Hubei, where the first case of COVID was detected, the city was not only experiencing the highest infection rate but also a high death rate. Logic would lead to one conclusion: that China would fail in its recruitment plan. However, against the odds, China has successfully recruited 42,000 medical workers nationwide, comprising medical doctors and nurses coming to Wuhan Hubei [4]. Among them, the first batch of medical workers arriving from Jiangsu province, a region that was considered to be less infectious according to the *Circular on the adjustment of COVID-19 classification standards and implementation of precise control measures* by the State Council, comprised 147 volunteers. In accordance with the unified deployment of the National Health Commission, the hospital issued a recruitment notice to support Wuhan in the fight against the COVID-19 pandemic, and volunteers from medical doctors to nursing staff responded. At the same time, the hospital urgently prepared medical supplies and daily necessities for healthcare professionals, so that healthcare professionals could complete front-line work without worries. Research that was originally conducted to explore the experiences of the healthcare professionals has been expanded to gain better insight into the unexpected responses of these healthcare professionals to the recruitment drive. Particularly for the first batch of medical workers to Wuhan arriving as support, they would face greater risks and more uncertainty.

Studies revealed the high prevalence of depression and anxiety among nurses during the COVID-19 pandemic [4,6,7,8,9,10,11]. These negative emotional states were attributed to a host of negative experiences, included but not limited to “not knowing about the disease”, “inadequate personal protective equipment” and “low self-efficacy as a healthcare professional” [11,12,13,14,15]. In some cases, workload and family relationships were also found to have caused depression and anxiety during the pandemic [6,7,15,16,17]. Among all the possible reasons for this, the fear of contracting COVID-19 in the course of providing care to patients with COVID-19 was also unanimously reported in studies that specifically explored the experiences of healthcare professionals who rendered care to COVID victims. Certainly, experiences were found to have a significant impact on healthcare providers’ mental health [18,19,20,21]. Such findings were perhaps not surprising, because COVID-19 infections among healthcare professionals have been estimated at more than 150,000 in Europe alone, with over 620 deaths [6]. China also saw over 1700 infections among their healthcare professionals, with close to 6 deaths. Healthcare professionals’ fears of contracting COVID were certainly legitimized by a study in Australia that concluded that healthcare professionals who cared for COVID-19 victims were 2.76 times more likely to contract COVID-19, regardless of their area of work [22]. This might explain why there was no difference in the prevalence of depression between nurses who worked in high-risk COVID-19 wards and those working in low-risk COVID-19 wards [6]. In the context of these known fears and negative emotions among the healthcare professionals, as part of a larger study, the current analysis was conducted to explore the most plausible reasons for China’s successful recruitment of healthcare professionals from the least-infected region to the highly infectious Wuhan area. The aim was to gain insight into the decision to help, made by these volunteers.

## 2. Materials and Methods

### 2.1. Research Design

A qualitative interpretative phenomenological analysis (IPA) was used to provide the theoretical framework for the current study. IPA was developed by Jonathan Smith [23,24,25]. The fact that IPA places central importance on the individual meanings ascribed to salient experiences makes it an optimal research methodology to explore the subjective experience of healthcare professionals from a very low-infection-rate area in China, who have responded to the invitation by the Chinese National Health Commissioner to render care to the victims in the highly infectious area of Wuhan [26]. 

### 2.2. Settings and Participants

This research was conducted at three grade-A tertiary hospitals (Subei People’s Hospital of Jiangsu province, Affiliated Hospital of Yangzhou University, and Yangzhou Hospital of Traditional Chinese Medicine) in Yangzhou where the recruits were employed. We used the IPA approach, which focuses on small and homogeneous samples [27]. Participants were recruited on the basis of their personal experience and views. The snowball sampling was based on the solicitation of healthcare professionals who had agreed to participate in this interview, in order to attract other participants with similar experiences to join the research [28]. Therefore, using purposive and snowball sampling, healthcare professionals were invited to help establish reasons for healthcare professionals to make the move to Wuhan, when they could remain “safe” in low-risk regions. The saturation criterion was adopted to determine the sample size, which is an acceptable method to estimate sample size [29]. Letters of invitation explaining the purpose of the study and contact details of the second and third researchers were sent out to the three hospitals mentioned above in Yangzhou, Jiangsu province, China.

### 2.3. Data Collection

Since IPA is conducted with a detailed account of individual experiences, every healthcare professional involved in this research reflected on their experience of supporting Wuhan from their own point of view. The interview outline (Table 1) was constructed by P.T.Z. and J.L. Basing the meetings on the participants’ choice of date, time and place, in-depth semi-structured interviews in a face-to-face context were conducted from 1 September 2020 to 14 November 2020. Before data collection, researchers tried to build rapport by asking participants about their daily lives. At the same time, a non-threatening and comfortable environment was created for each interview. When the interview began, health care workers were asked to talk step-by-step about their experiences, from signing up for service to their return to Yangzhou. Interviews were conducted in Chinese and audio-recorded and ranged in length from 74 to 95 min. Participants could terminate their participation at any time and, if they suffered mood swings, they would be counseled by a researcher qualified as a psychological consultant until the mood lifted. In this research, none of the healthcare professionals contacted terminated their participation. The transcripts were generated verbatim in the participants’ spoken Chinese language and were then translated into English. The transcripts were translated back into Chinese for participation validation. To ensure credibility and confirmability, the forward and backward translation processes were repeated; this process established two interims of translation completion, at which point participant validation was conducted. Participant validation then ended, and data analysis commenced when the researchers were confident that the actual meanings by all participants were preserved in the translation. 

### 2.4. Data Analysis

Data analysis began with a close examination of the first case, leading to the development of case themes, and then we considered recurring themes across the dataset [28]. Data analysis occurred in six stages, the details of which are described in Table 2. In stage 1, the researchers read the data and transcript of a single case; in stage 2, the researchers recorded their observations in the margins of the transcript; in stage 3, the researchers developed emergent themes based on data related to the observation notes; in stage 4, the researchers explored the connections between themes by considering the relationship between “data” and “observation notes”. After completing this case study, the researchers moved on to the next case, repeating stages 1–4 for each case. In stage 5, the researchers identified recurring themes in these cases and noted down any particular differences. In stage 6, the researchers reviewed the themes through the data set, aiming to elicit further meaning from the experience [23]. The themes given below were derived from interviews with more than 50% of the participants, and the frequency and content of the presentation of themes are shown in the Supplementary Materials.

### 2.5. Rigor

During the interview, the researcher listened and observed carefully, asked and responded to valuable questions appropriately, used techniques such as repetition and response, observed the tone, facial expressions and body movements of the interviewees, and took notes in time to ensure credibility of the data. The corresponding authors received professional training in qualitative methodologies and were familiar with the unique challenges posed by qualitative approaches to such an inquiry. At the same time, throughout the analysis of the data, the research team sought to ensure that participants’ experiences were sensitively reflected and that the analytical process was transparent and rigorous. The researchers discussed what the participants really meant by the experiences they described and tried to interpret the participants’ views in new ways.

### 2.6. Ethical Approval

Confirmation of the ethical review was obtained from the Nursing School at Yangzhou University (Code YZUHL2020002). Permission to conduct the study was obtained from the hospitals, and informed consents were obtained from the participants. Participants were informed that they could terminate the interview at any time, should they feel distressed. A debriefing sheet that detailed a list of support services was provided for each participant.

## 3. Results

Four medical doctors and ten nurses provided informed consent and participated in the study. Participants ranged in age from 28 to 41 years. Three nurses and one medical doctor were from the “Subei People’s Hospital of Jiangsu province”. Four nurses and one medical doctor were from the “Affiliated Hospital of Yangzhou University”. Other participants were from the “Yangzhou Hospital of Traditional Chinese Medicine” (Table 3). Of the fourteen participants, only three were single. Other than one nurse who lived alone, the rest were residing with their families, and all had dependents at home. All participants received their formal education in China; this included their mainstream education and their undergraduate and postgraduate professional degree programs. All had more than five years of experience as healthcare professionals.

All participants reported feeling fearful at some point regarding their volunteered professional services at Wuhan. While some were only worrying about being infected, they suffered from insomnia the night before they left for Wuhan:

“I didn’t sleep well the night before I left.” (N2)

The fear of not being able to return to their families ran through some of the participants’ minds. Many had spoken to their next of kin as if it was the last time they would be speaking with them.

“…I was afraid when I went, I told my husband the code of my pay card” (N1)

“…in case I get infected, tell me something …” (MD1)

Despite the fear of infection and possible death from this trip, all of them had persisted and had fulfilled their agreement to render care and support at Wuhan. No one had given up halfway, and all had completed the mission before returning to Yangzhou on the originally agreed date.

The accounts of the participants explained their reasons for the various mixed feelings of the individuals at different stages of their voluntary work at Wuhan. These are categorized in three primary superordinate themes, which the participants recounted as: “Trusting the Chinese health authorities”, “Justifying personal actions and decisions” and “Negotiating and reclaiming identities”. We will now discuss these findings in detail.

### 3.1. Theme 1—Trusting the Chinese Health Authorities

We named the first thematic cluster within the first category, “Practical aspects of stressors linking to home—well-managed by the local Chinese authorities”. The belief among the 14 participants that “health authorities are managing everything effectively” conveys the importance of the individuals’ trust that they place in the Chinese authorities, and more importantly, their expectations, plans and feelings as they prepared for their journey to Wuhan.

They could also readily trust the authorities because, from their perspective, the authorities were seriously concerned about their health status; during their stay at Wuhan, gowns, masks, and alcohol were abundantly available for their use, and there was good mentorship to boost their morale. In addition, they were extremely pleased to have received abundant good-quality food and prophylactic Chinese medication, which to them was special treatment:

“… the traditional Chinese medicine [is] given by the Yangzhou Hospital of Traditional Chinese Medicine… [the] Jiangsu provincial government also gives us thymosin injections to enhance immunity. One injection of thymosin costs more than 300 yuan; we have two injections a week.” (N1)

“…my hospital prepared traditional Chinese medicine to prevent colds for us before departure, which had an effect on invigorating the body and replenishing qi.” (N2)

“There was no heated blanket in Wuhan. And then our hospital leaders sent it to where we live in Wuhan because it’s freezing cold.” (N9)

“The supplies… for us were brought over by Jiangsu Province.” (MD2)

The second thematic cluster identified was “Practical aspects of stressors linking to work: lack of PPE, fear of infection—evidently well-managed by the national Chinese health authorities”. The medical doctors and nurses supporting Wuhan were well protected by taking precautions, maintaining social distancing, and providing adequate protective materials.

“Generally, two people wash [their] hands together, far away from each other.” (N1)

“We usually work five hours a day and we work a day and then take a day off in Wuhan. In Wuhan, it is necessary to report our temperature every morning.” (N5)

“... we had to line up outside the door and waiting to get off work one after another, we were separated by 1 m when we were in line.” (N8)

The third thematic cluster identified was “Psychological aspects of stressors linking to work: uncertain, high-risk and heavy workload—evidently well managed by the national Chinese health authorities”. The government and hospitals provided adequate organizational support and various forms of psychological support to relieve healthcare professionals’ psychological pressure.

“...we got some psychological support because our mental health is also very important.” (N2)

“When we are under pressure, we… communicate with our colleagues.” (N4)

“The group has organized psychological counseling, such as Balint group, which conducts psychological interventions for health care workers.” (MD2)

The fourth thematic cluster identified was “Good physical health maintained and preserved by the national Chinese health authorities”. Each participant thanked the authorities for the guarantee of their physical health, not only in terms of life but more importantly, for the humanistic care provided to them.

“I was really grateful that all of the treatment costs and the food were borne by our country.” (N8)

“The leader of the medical team also gave us personalized rest for the special period, for example, he would arrange [for] me to have a rest if I feel uncomfortable when I experienced menstruation, he cared a lot about us.” (N10)

“My routine in Yangzhou is four days in every shift round, day shift is 8 h, night shift is 16 h then I take one day off. In Wuhan, I work for 4 h and had 24 h for rest.” (MD3)

### 3.2. Theme 2—Justifying Personal Actions and Decisions

The second category of themes consisted of two clusters: “Social support and benefits to families of volunteers” and “Professional recognition and development”.

Participants emphasized the subjective experience as being like a family who could trust the health authorities for any help and support, including their children’s education, parents’ health, and family financial welfare support.

“The government delivered some daily necessities, such as food. For my family, the government gave 2000 yuan. When I was in Wuhan, there was [a] government subsidy, … It is remitted to my bank card.” (N3)

“Some third-party network platforms have given us thousands of yuan… as consolation money. The teachers of [my] children’s school have come to our home to express their sympathy.” (N5)

“The Women’s Federation, social organizations cared a lot [for] my family and some training institutions for children which provided discounts to us.” (MD3)

Both physicians and nurses have felt their professional value has grown in supporting COVID-19 treatment in Wuhan. They gave recognition to their own value and put their knowledge and skills into practice. They thought it was a part of that recognition that they had the opportunity to go to Wuhan to support the COVID-19 outbreak.

“I feel that the profession of nursing can really help people and the knowledge of our profession can also be… very useful.” (N5)

“Nursing is a hard job… [in] itself, but it gives me a sense of value.” (N10)

“Everyone got rewards according to personal performance.” (MD2)

### 3.3. Theme 3—Negotiating and Reclaiming Identities

The first cluster identified was “Conceptualization and externalization of social role—accepting imperfections in the social role”. Participants mentioned that in their social roles, they had some family responsibilities that they did not fulfill before they went to Wuhan. Finally, feeling the support of their family members, they rushed to the front without hesitation.

“It didn’t have a big impact because I had been studying outside for years. My daughter was raised by my parents, and my husband also supported me to go to Wuhan.” (N1)

“My husband was in charge of my child’s daily life when I was at Wuhan.” (N8)

“When I was in Wuhan, my mother-in-law had taken my place.” (N9)

The second cluster identified was “Conceptualization and internalization of the professional role—highlighting professional obligations”. As seen in each participant interview, they all stated their love for their profession as health care workers. They all considered it their professional responsibility to go to the front line to support the treatment of COVID-19 patients.

“What motivated me to go is the love of my profession.” (N1)

“I think there is no reason for me to flinch because I am a nurse and I [am] supposed to face it. This is the spirit and the moral of the profession.” (N2)

“I never thought that I might get some honor or some benefits for my career in the future, neither did I think that I would get anything in return when I came back.” (N10)

The third cluster identified was “Conceptualization and internalization of social identity as a Chinese National”. The decision of Chinese health care workers to go to Wuhan was influenced by Chinese culture. In the face of disasters and epidemics, Chinese health care workers have often chosen to sacrifice their own interests to protect the interests of others and the country.

“I think as a health care worker, when the country needs you, you have to abandon your family and personal interests.” (N3)

“Anyway, under such circumstances, once the interest of individuals and collectives were at odds, consideration must be given to the whole team.” (N4)

“We twisted ourselves into a rope and [are] moving… in a better direction.” (MD2)

## 4. Discussion

The emotional state of feeling depressed and anxious and the negative experiences were separate entities, which of course may well have co-existed during this pandemic. It is important to know that the negative experiences from the pandemic may have preceded the development of the later negative emotional states; they may well have developed as a consequence of that particular health care professional feeling anxious and depressed even before they volunteered. It was, therefore, important to explore the experiences of medical doctors and nurses who volunteered to render care in Wuhan during its highly infectious period.

This study aimed to achieve a better insight into China’s successful recruitment, to give better preparation in a similar health crisis on a scale as large as the current pandemic. Despite the geographical differences, in this secular world where personal safety and personal interests were increasingly the focus of life, logically, personal safety would be a priority for any nurses and medical doctors, and this would be even more important during this pandemic. Some of the healthcare workers who were persistent in their professional ethics during the fight against COVID-19 may have added burdens of worries that were not only related to their front-line work but were also directly linked to their being homesick.

The uncertainty of COVID-19 has caused tremendous stress and distress for healthcare professionals [30]. When the recruitment notice was issued, Wuhan’s local medical resources and human resources were seriously lacking, and healthcare professionals were facing a situation where there was not enough personal protective equipment (PPE). In other countries, media reports documented nurses’ frustration over the failure of their government to do everything possible to keep frontline health care professionals safe [31]. In the United States, nurses were angry at the government’s lack of PPE supply for health care professionals [32]. However, under such circumstances, the Chinese government can meet the requirements of health care professionals in a timely manner, such as providing adequate PPE and reasonable scheduling. The Chinese government and healthcare professionals have made great efforts to optimize workflow and ensure the proper use of protective equipment, cleaning and disinfection measures. In addition, the Chinese government can provide timely psychological support and crisis intervention to healthcare professionals, including psychological assistance for typical problems, one-on-one psychological assistance, and relaxation training, to alleviate negative emotions among healthcare professionals, and to affirm their own value and professional recognition [33].

Before going to Wuhan, healthcare professionals relied on their family and friends as a support system. When their support systems were deprived through isolation, the support of government and leaders had to be provided [34]. Another effective approach was to encourage colleagues to quickly build more connections and support. In the meantime, when healthcare professionals were on the front line, they were unable to be with their families and fulfill their family roles, so they felt guilty and worried about their families’ health [21]. The Chinese government pays timely attention to the families of front-line healthcare professionals. If their families are in difficulties, it is necessary to report to the coordinating management to resolve the family difficulties and give healthcare professionals peace of mind to do their job [33].

We found that healthcare professionals in respiratory, infectious, and critical care departments, who had experience fighting infectious or respiratory diseases, were less psychologically stressed than those in some other departments because they had more confidence in their abilities. This finding is consistent with previous studies [35], showing that experienced and competent healthcare professionals will be ready to work during future pandemics.

It is worth mentioning that this study provided us with a better insight into the professional ethics of nurses and medical doctors. Healthcare professionals have volunteered their time and effort to help at the front line, regardless of the risks. This research helped us better understand how health care workers could make the decision to join a task force that others would have wanted to stay away from.

Just slightly more than a decade ago, when the concept of professional values was still relatively new in China, Pang et al. had already identified seven professional values among the Chinese nurses: namely, altruism, caring, trustworthiness, dignity, responsibility for the development of the profession, autonomy, and justice [36]. These values were consistent with the Code of Ethics for Nurses set by the International Council of Nurses (ICN) and the Code of Ethics for Nurses set by the Chinese Nursing Association (CNA) [36]. As for the medical doctors in China, their professional values are embedded in the Chinese medical ethics tradition [36]. In the Chinese language, the emphases in the field of ethics were “jing” in relation to medical skills and “cheng” in relation to the patient. Using a direct translation, “jing” simply means refinement and “cheng” means sincerity. However, “jing” is much more profound than “refinement”, and “acumen” and “cheng” is much more than merely “sincerity” but also “honesty”, “transparency”, and, to a large extent, a promise of commitment between the doctor and his patient. That commitment is the doctor‘s capability in fulfilling what he agreed (with his patient) with regard to his medical treatment plans [36]. These two cardinal values—the notion of the highest-quality medical care with a strong sense of loyalty in protecting the patients—are expected of every medical doctor.

Despite the slight differences in how professional ethics are inculcated in the two healthcare professions, those individuals who were enrolled in any undergraduate programs to pursue medicine or nursing were unavoidably subject to a mandatory course on Mao Zedong’s ideas. “Rescue the dying, heal the wounded, and serve the people wholeheartedly”, are the critical principles within the ethical mandate that are instilled in each and every nursing and medical student, right at the start of their pursuit of a career as a healthcare professional in a Chinese higher education institution. Participants’ accounts have also provided a good insight into healthcare professional ethics, acting as their stable and strong pillar of support to the noble work for which they volunteered so willingly and readily.

There exist some limitations in this study. First, it was conducted only among healthcare professionals in Yangzhou city, which limits the representativeness of the samples and the generalizability of the results. Second, quantitative approaches that are employed to measure anxiety and depression in healthcare professionals did not act as components of this study. At the same time, when conducting research related to COVID-19, the experience in Wuhan should be considered as a stressor for participants, and appropriate emotional support should be given.

## 5. Conclusions

Despite the limitations of this research, it provides a better understanding of the nature of healthcare professional ethics during a crisis. The current study focuses on the applicability of these ideas during the recent pandemic, in the hope of providing a plausible explanation for the success of the recruitment drive for healthcare professionals to the worst-hit city of Hubei during the pandemic. This study provided us with a better insight into the reasons for the various mixed feelings of the volunteers during the work at Wuhan. It has also gleaned a better understanding of healthcare professional ethics, as a stable and strong pillar of support for their persistence. In future pandemics and public health events, governments and medical institutions should prioritize the health, safety and well-being of healthcare professionals when recruiting volunteers to support in high-risk areas. Healthcare professionals should be provided with a healthy workplace to enhance their professional identity. Most importantly, there is a need to raise awareness of the personal and professional values of healthcare professionals and to make the most helpful decisions for their situation.

## Figures and Tables

**Table 1:** Interview schedule outline.

What was your inner motivation when you decided to support Wuhan?
Did you discuss the topic with your family when you made this decision?
What made you decide to go to Wuhan; why did you want to go there?
What runs through your mind when you sign up to be a front-line healthcare professional?
Can you tell me the whole process of registration and your mental process?
Can you talk about your feelings when you were on your way to Wuhan?
What ran through your mind when you reached Wuhan on that first day?
Can you tell me your feelings when you treat care patients with the COVID-19 virus?
Were you worried about contracting the COVID-19 virus at work?
Have your mind and emotions changed since you arrived in Wuhan?
After you participated in this rescue operation, do you have any new views on your occupation?

**Table 2:** Interpretative Phenomenological Analysis Steps.

Stages	Details
1. Reading and rereading	Researchers need to immerse themselves in the data. At the same time, using recordings made at the time, this provides some new insights into the data.
2. Initial noting	Researchers can jot down his or her observations and reflections on the interview experience, or any other thoughts and comments that might be meaningful. They may focus on content, language use, context, and initial interpretative comments.
3. Developing emergent themes	At this stage, researchers should study and analyze the “observation notes”. The purpose of this stage is to transform the “observation notes” into “emergent themes”.
4. Searching for connections across emergent themes	This stage involves looking for connections between emergent themes, grouping them based on conceptual similarity, and providing a descriptive description for each cluster. Some themes may be removed at this stage if they do not match the emerging structure, or because their evidence basis is weak.
5. Seeking patterns across cases	Researchers kept asking, “Are there identifiable themes in different cases? It’s useful to focus on different experiences and emotional reactions.”
6. Changing the interpretation to a deeper level	Researchers reviewed the themes through the data set, aiming to elicit further meaning from the experience.

**Table 3:** Characteristics of the study participants.

Healthcare Professionals(HCP)	Age	Gender	Marital Status	Family Profile (Year)	Years as an HCP	Professional Title/Role	Specialties	Hours Working Per Week (Average)
MD1	41	Male	Married	Mother (68) Wife (40) Child 1 (13) Child 2 (3)	17	Deputy Medical Director	Accident and Emergency	>72 (28 in Wuhan)
MD2	40	Male	Married	Wife (40) Child 1(12) Child 2(3)	17	Deputy Medical Director	Respiratory Department	40 (32 in Wuhan)
MD3	38	Male	Married	Mother (61) Father (60) Wife (32) Child (5)	14	Medium-grade professional title	Internal medicine, and critical care	>48 (16 in Wuhan)
MD4	32	Female	Married	Mother (60) Mother-in-law (63) Husband (37) Child (2)	6	Medium-grade professional title	Respiratory Department	>42 (12–18 in Wuhan)
N1	33	Female	Married	Father (56) Mother (56) Husband (37) Child 1 (7) Pregnant with second child (when in her LMP)	12	Nurse-in-Charge	General surgery	40 (16 in Wuhan)
N2	32	Female	Single	Mother (64)	8	Senior Nurse	Intensive Care	40 (16 in Wuhan)
N3	39	Female	Married	Mother-in law (62) Husband (41) Child 1 (12)	17	Nurse-in-Charge	Infection Control: 15 years	50 (16 in Wuhan)
						Nurse-in-Charge	Traditional Chinese medicine: 2 years	
N4	35	Female	Married	Husband (35) Child 1 (10) Child 2 (3)	12	Nurse-in-Charge	Respiratory Department	40 (16 in Wuhan)
N5	35	Female	Married	Mother (59) Husband (36) Child (12)	15	Nurse-in-Charge	Respiratory Department	40 (20 in Wuhan)
N6	28	Female	Single	Father (51) Mother (50)	6	Senior Nurse	Traditional Chinese medicine	40 (16 in Wuhan)
N7	29	Female	Single	Live alone	7	Senior Nurse	Emergency and intensive nursing	>48 (18 in Wuhan)
N8	40	Female	Married	Husband (42)Child (13)	18	Head Nurse	Neurosurgery	>50 (16 in Wuhan)
N9	38	Female	Married	Husband (42) Child (11)	18	Nurse-in-Charge	Surgical nursing	40 (16 in Wuhan)
N10	30	Female	Married	Husband (30) Child (3)	7	Nurse-in-Charge	Neurosurgery	>45 (16 in Wuhan)

MD: Medical doctor; N: Nurse.

## Data Availability

The data that support the findings of this study are available from the corresponding author upon reasonable request.

## Supplementary Materials

The following are available online at https://www.mdpi.com/article/10.3390/ijerph18168737/s1, Table S1: Themes for All Fourteen Participants Illustrated with Quotations.
