# Supplementary material for: China’s Successful Recruitment of Healthcare Professionals to the Worst-Hit City: A Lesson Learned
Source: Int J Environ Res Public Health. 2021 Aug 19;18(16):8737. doi: 10.3390/ijerph18168737 (PMC8393906; doi:10.3390/ijerph18168737)
Supplement: Supplementary file 1 [file ijerph-18-08737-s001.zip › ijerph-1312491-supplementary.pdf]

Table S1. Themes for All Fourteen Participants Illustrated with Quotations.

Participant number	Quotations
Theme 1 - Trusting the Chinese health authorities	
First cluster: "Practical aspects of stressors linking to home - would be well manage by the Local Chinese Authorities	
N1	"… the traditional Chinese medicine given by the Yangzhou Hospital of traditional Chinese Medicine… Jiangsu provincial government also gives us thymosin injection to enhance immunity. One injection of thymosin costs more than 300 yuan, we have two injections a week."
N2	"…my hospital prepared traditional Chinese medicine to prevent colds for us before departure, which had an effect on invigorating the body and replenishing qi."
N5	"The director of our nursing department told us that we could ask them for help, if my family experience any difficulties in Yangzhou."
N6	"I thought my hospital has done its best. Any kinds of supplies that we need, they all sent over. There was a time, we asked some protective socks. Then our hospital sent a lot of socks to us."
N9	"There was no heater blanket in Wuhan. And then our hospital sent the it to us, because it's freezing cold in Wuhan."
N10	"Our hospital provided us with a lot of drugs, such as antidiarrheal drugs, cold drugs, antiviral drugs and vitamin C, we inject thymosin every three days. In addition to prepare medicine for us, our hospital also prepared heater blankets for us. Because it was quite cold at that time."
MD2	"Video meetings were held every night to discuss the disease, including the meeting with Yangzhou medical team and the meeting with the whole medical staff from Jiangsu province."
Second cluster:" Practical aspects of stressors linking to work: lack of PPE, fear of infection - evidently was well manage by the National Chinese Health Authorities"	
N1	"Generally, two people wash hands together, far away from each other."
N3	"The standard of COVID-19 was changed keep pace with the times by our country, the leader of us asked some outside expert to teach us some knowledge about this virus."
N5	"We usually work five hours a day and we work a day and then take a day off in Wuhan. In Wuhan, it is necessary to report our temperature every morning."
N8	"... we had to lined up outside the door and waiting to get off work one after another, we were separated by 1 meter when we were in line."
N9	"Such as how to put on and take off the protective suit and how to wear gloves. These processes must be standardized and we train over and over again."
N10	"…gave us personalized rest for the special period, for example, he would arrange me to have a rest if I feel uncomfortable when I experienced menstruation, he cared a lot about us."
MD2	"We wore protective suit and protective goggles when we worked."
Third cluster:" Psychological aspects of stressors linking to work: uncertain, high risk and heavy workload- evidently was well manage by the National Chinese Health Authorities"	
N2	"...we got some psychological support because our mental health is also very important."
N3	"I'm not afraid at all. I'm with people from our hospital. We have leaders in each group."
N4	"When we are under pressure, we will communicate with our colleagues."
N6	"But for me, um ... (silent for a while), it will still be a little stressful, we will read the relevant nursing guides carefully, and we need to be well protected."
N10	"I asked a psychologist to help me, and then I got better."
MD 2	"The group has organized psychological counseling, such as Balint group, which conducts psychological interventions for health care workers."
MD 4	" The leaders of hospital took much count of us and cared us a lot."
Fourth cluster:" Good physical health maintained and preserved by the National Chinese Health Authorities."	
N1	"Our country's policy is really good, the food and drink arranged for us are good."
N3	"I think the Chinese government handled the epidemic in a very timely manner."
N8	"I was really grateful that all of the treatment costs and the food were borne by our country. "
N9	"Once I become an asymptomatic infected person, my country will protect me. I will be isolated and receive treatment correctly"
N10	"The leader of the medical team also gave us personalized rest for the special period, for example, he would arrange me to have a rest if I feel uncomfortable when I experienced menstruation, he cared a lot about us."

MD3	"My routine in Yangzhou is four days in every shift round, day shift is 8 hours, night shift is 16 hours then I take one day off. In Wuhan, I work for 4 hours and had 24 hours for rest."
MD4	"we've received concern from all over the country through Internet and we also received some consolation letters and material supplies."
Theme 2 - Justifying personal actions and decisions	
First cluster:" Social support and benefits to families of volunteers"	
N3	"The government delivered some daily necessities, such as food. For my family, the government gave 2000 yuan. When I was in Wuhan, there was government subsidy, … It is remitted to my bank card."
N5	"Some third-party network platforms have given us thousands of yuan of consolation money. The teachers of children's school have come to our home to express their sympathy."
N6	"The community and the hospital gave some condolences to my family, such as food and some disinfection supplies. Yangzhou Municipal Government gave us a subsidy of several thousand yuan and paid us for working at Wuhan."
N9	"Our hospital and Women's Federation went to my home to give condolences. My department of our hospital arranged a person to condolence my family."
N10	"Hospital leaders brought some vegetables and fruits to my home for condolences twice, the government also visited my home once, and they bought toys for my child. "
MD3	"The Women's Federation, social organizations cared a lot to my family and some training institutions for children which provided discounts to us."
MD4	When I stayed at Wuhan, my family was consoled by some foods, vegetables, fruits and so on. And I was so touched.
Second cluster:" Professional recognition and development"	
N1	"I think it's worthwhile for our medical staff to go to Wuhan to help them in this critical time in this country. It's a reflection of our value."
N2	"It felt a sense of accomplishment in severe illness, because we pulling the patient back from the death line."
N5	"I feel that the professional of nursing can really help people and the knowledge of our professional can also be used very useful."
N7	"The nurse plays a big role in the fight against COVID-19. Nursing is not a profession to be looked down upon."
N10	"Nursing is a hard job for itself, but it gives me a sense of value."
MD2	"Everyone got rewards according to personal performance"
MD3	"In the later period, there are fewer and fewer patients in the isolation ward and it was gradually emptied, at the same time, we also see the hope. From being overwhelmed and fearful to finally completing the task and feeling proud."
Theme 3 -Negotiating and Reclaiming identities	
First cluster:" Conceptualisation and externalisation of social role - Accepting imperfections in social role"	
N1	"It didn't have a big impact because I had been studying outside for years. My daughter was raised by my parents, and my husband also supported me to go to Wuhan."
N2	"Afterall, I am the only child of my parents."
N4	"The first reason I didn't want to go Wuhan was that my children were too young and my parents were old."
N5	"I just need my family's support. When my husband promised me that he would take care of the family, I was more convinced of my decision."
N8	"My husband was in charge of my child's daily life when I was at Wuhan."
N9	"When I was in Wuhan, my mother-in-law had taken my place."
MD1	"My wife and I are both doctors, and I'm an emergency department doctor."
Second cluster: "Conceptualisation and internalisation of professional role – highlighting professional obligations"	
N1	"What motivates me to go is the love of my profession."
N2	"I think there is no reason for me to flinch, because I am a nurse and I suppose to face it. This is the spirt and the moral of the profession."
N4	"Due to my profession, the spirit of the profession."
N8	"At this time, we had no choice and had to clean their feces up and sucked sputum. But this is my duty."
N9	"As a medical worker, we should have professionalism and a sense of responsibility to get things done. And we should love our own profession."
N10	"I never thought that I might get some honour or some benefits for my career in the future, neither did I think that I would get anything in return when I came back."

MD3	"Although it was very tired when I worked at Wuhan, but it was worth it."
Third cluster: "Conceptualisation and internalisation of social identity as a Chinese National"	
N3	"I think as a health care worker, when the country needs you, you have to abandon your family and personal interests desperately"
N4	"Anyway, under such circumstances, once the interest of individual's and collectives' were at odds, consideration must be given to the whole team."
N7	"All of the measures epidemic has shown that our country will not give up anyone as long as there is hope. China is so powerful not only in technology, but also the power of unity. Once we are united, I can feel the power and I have confidence in that."
N9	"Obeyed the arrangement like in an army and I would go everywhere once my country need me."
N10	"Because it happened in China and we all Chinese, that is the reason why we were working together to defeat the virus."
MD2	"We twisted ourselves into a rope and moving to a better direction."
MD4	"We all tried so hard and united because we have the same aim."
